# Critical role for BIM in T cell receptor restimulation-induced death

**DOI:** 10.1186/1745-6150-3-34

**Published:** 2008-08-20

**Authors:** Andrew L Snow, João B Oliveira, Lixin Zheng, Janet K Dale, Thomas A Fleisher, Michael J Lenardo

**Affiliations:** 1 Molecular Development Section, Laboratory of Immunology, National Institute of Allergy and Infectious Diseases, Bethesda, MD, 20892, USA; 2 Department of Laboratory Medicine, Clinical Center, National Institutes of Health, Bethesda, MD 20892-1508, USA; 3 Laboratory of Clinical Infectious Diseases, National Institute of Allergy and Infectious Diseases, National Institutes of Health, Bethesda, MD, 20892, USA

## Abstract

**Background:**

Upon repeated or chronic antigen stimulation, activated T cells undergo a T cell receptor (TCR)-triggered propriocidal cell death important for governing the intensity of immune responses. This is thought to be chiefly mediated by an extrinsic signal through the Fas-FasL pathway. However, we observed that TCR restimulation still potently induced apoptosis when this interaction was blocked, or genetically impaired in T cells derived from autoimmune lymphoproliferative syndrome (ALPS) patients, prompting us to examine Fas-independent, intrinsic signals.

**Results:**

Upon TCR restimulation, we specifically noted a marked increase in the expression of BIM, a pro-apoptotic Bcl-2 family protein known to mediate lymphocyte apoptosis induced by cytokine withdrawal. In fact, T cells from an ALPS type IV patient in which BIM expression is suppressed were more resistant to restimulation-induced death. Strikingly, knockdown of BIM expression rescued normal T cells from TCR-induced death to as great an extent as Fas disruption.

**Conclusion:**

Our data implicates BIM as a critical mediator of apoptosis induced by restimulation as well as growth cytokine withdrawal. These findings suggest an important role for BIM in eliminating activated T cells even when IL-2 is abundant, working in conjunction with Fas to eliminate chronically stimulated T cells and maintain immune homeostasis.

**Reviewers:**

This article was reviewed by Dr. Wendy Davidson (nominated by Dr. David Scott), Dr. Mark Williams (nominated by Dr. Neil Greenspan), and Dr. Laurence C. Eisenlohr.

## Background

Proper homeostasis is achieved *during *an immune response by controlling the appropriate size and activity of the effector T cell pool to maximize immunity and minimize immunopathology. *After *an immune response, homeostasis depends on the efficient contraction of the expanded T effector pool. Both processes require the selective death of effector T cells [1-5]. When resting T cells become activated and proliferate under the influence of growth cytokines, they display heightened sensitivity to apoptosis [3,5,6]. The mechanisms by which apoptosis is provoked have been thought to differ depending on the level of antigen in the T cell milieu. In one simple schema, T cell apoptosis proceeds through either an "intrinsic" (Bcl-2 superfamily/mitochondrial-dependent) program when antigen levels are low, or an "extrinsic" (Fas/CD95/APO-1/death receptor-mediated) pathway under conditions of high or repeated antigen stimulation [1,4,5,7]. In the first case, antigen clearance at the conclusion of an immune response results in diminished growth and survival cytokines (such as interleukin-2 (IL-2)), thus activating the mitochondrial death program. Cytokine withdrawal apoptosis (CWA) dramatically reduces the expanded T effector population to re-establish homeostasis, but permits a small population to persist as memory T cells. CWA is principally regulated by the pro- and anti-apoptotic members of the Bcl-2 family. In particular, the pro-apoptotic "BH3-only" proteins Bim and Puma have been implicated in CWA, as revealed by expanded memory T cells in knockout mice [8-10]. These "BH3 only" members of the Bcl-2 superfamily cause caspase activation and apoptosis by binding pro-survival congeners and releasing the proapoptotic proteins Bax and Bak [11]. Moreover, we recently discovered that a gain-of-function mutation in N-RAS, which suppresses Bim expression via constitutive extracellular signal-related kinase (ERK) activation, could cause a novel form of ALPS in humans [12]. Indeed, Bim expression is tightly controlled by several transcriptional and post-translational mechanisms that underscore its role in central and peripheral T cell tolerance [13].

On the other hand, the extrinsic apoptosis pathway involves restimulation of activated T cells with high doses of antigen during the immune response; a pathway often referred to as "activation-induced cell death (AICD)" [1,7,14]. However, it is important not to obfuscate the critical functional distinction between "activation" – the process entrained to the antigen receptor that causes resting cells to cycle, expand, and acquire effector function – and the death mechanism induced by TCR restimulation of those effector T cells that counterposes their expansion. So the term "TCR restimulation" or "TCR-induced" apoptosis will be used herein. The key immunoregulatory consideration is why restimulation by same antigen that produced the immune response, can kill the participating T cells in a highly specific way. At first glance, this event would seem to debilitate the immune response since the antigen, and presumably its pathogenic source, are still present. However, it is best understood as a negative feedback mechanism that constrains effector T cell proliferation to avoid immunopathology, previously termed "propriocidal" regulation [3,4,6]. Propriocidal or TCR-induced death increases proportionately with high or persistent levels of antigen in IL-2. TCR-induced death has hitherto been primarily equated with the Fas death receptor. Indeed, the upregulation of Fas ligand (FasL) on the surface of restimulated T cells engages Fas on effector T cells in *cis *("suicide") or in *trans *("fratricide") leading to apoptosis [15-18]. Moreover, debilitating mutations in Fas or FasL result in defective lymphocyte homeostasis and autoimmunity first characterized in mice (*lpr *and *gld*, respectively) and later in humans with ALPS type Ia or Ib [19,20].

The Bim vs. Fas paradigm recently restated for intrinsic vs. extrinsic T cell apoptosis is appealing in its simplicity but illusory. For instance, other BH3-only proteins such as PUMA are likely instrumental in CWA [9,10]. Also, the evidence suggests that Fas may not be the sole mediator of TCR-induced death and that TNF or nonapoptotic pathways may be involved [21,22]. Data from conditional knockout mice in which Fas is ablated or blocked in distinct hematopoietic compartments indicate that Fas-mediated apoptosis may also counter autoimmunity by ensuring the removal of antigen presenting cells, including B cells and dendritic cells rather than T cells [23,24]. Although autoreactive T cells accumulate in T cell-specific Fas knockout mice, surprisingly, loss of Fas confers no selective survival advantage for T cells exposed to repeated antigen challenge [24]. Also, Fas engagement can intersect with the intrinsic pathway through a caspase-8 activating cleavage of Bid – a Bcl-2 superfamily member that can trigger mitochondrial apoptosis. Based on these insights, we asked whether death effector pathways other than Fas, including intrinsic signals routed through mitochondrial activation, were important for TCR-induced death of human T cells.

In re-examining human T cells in which FAS signaling is blocked or genetically impaired, we found that TCR-induced apoptosis can proceed through rapid induction of BIM expression in the absence of FAS signals, which contributes to mitochondrial permeabilization and cell death in the presence of IL-2. Knockdown of BIM expression partially rescued cells from TCR-induced death, particularly for CD8^+ ^human T cells. Moreover, we show that TCR-induced apoptosis is normal for ALPS Ia patients displaying elevated BIM expression, but impaired in an ALPS type IV patient in which BIM expression is repressed. Collectively, these data indicate that FAS and BIM can cooperate as independent effector molecules in TCR-induced apoptosis. Our results show BIM plays a key role in T cell contraction even when cytokines are abundant, indicating that FAS- and BIM-mediated T cell apoptosis are not mutually exclusive pathways as recently reinforced in the literature [7].

## Methods

### Cells and Treatments

Patients were enrolled and blood samples were obtained with informed consent under protocols approved by the National Institutes of Health (NIH). Peripheral blood lymphocytes (PBL) from normal donors were isolated by Ficoll density gradient centrifugation, and T cells were activated by either 5 μg/ml ConA or 1 μg/ml OKT3 mAb (Ortho Biotech, Raritan, NJ) plus 25 U/ml rhIL-2 (Peprotech, Rocky Hill, NJ), washed 3× in PBS, then cultured in 100 U/ml rhIL-2 for at least 7 days before apoptosis assays were performed. Activated T cell subsets were separated using CD4 or CD8 Microbeads and MACS magnetic bead cell separation (Miltenyi Biotec, Auburn, CA). In some experiments, inhibitors to caspase 8 (IETD-fmk) or caspase 9 (LEHD) (BioVision, Palo Alto, CA) were added at 20 μM. Caspase 9 enzymatic activity was measured using a Caspase 9 Colorimetric Assay Kit (BioVision) according to the manufacturer's instructions.

### Flow Cytometry

Apoptosis assays were performed as previously described [12]. Briefly, activated T cells were resuspended in fresh media + IL-2 and stimulated for 24 h with soluble OKT3 mAb, agonistic anti-Fas mAb APO1.3 (Alexis, San Diego, CA) plus 200 ng/ml Protein A, or 2 μM staurosporine. In some experiments, 1 μg/ml of an antagonistic Fas blocking Ab (clone SM1/23, Alexis) was added to cells 30 minutes prior to OKT3 restimulation. The level of apoptosis was determined by staining with 1 μg/ml propidium iodide and flow cytometry analysis using constant time acquisition as previously described. Mitochondrial permeability was measured by staining with 40 nM 3,3'-dihexyloxacarbocyanine iodide (DiOC6) (EMD Biosciences, San Diego, CA) for 15 min at 37°C before flow cytometry analysis. For surface staining, cells were stained with 5 μg anti-CD4-fluorescin isothiocyanate (FITC), anti-CD8-phycoerythrin (PE), or anti-CD95-PE (BD Biosciences).

### Electron Microscopy

Treated cells (5 × 10^6^) were pelleted and overlaid with 2% glutaraldehyde in 0.1 M cacodylate buffer fixative for 2 h at room temperature (RT). Sample preparation and electron microscopy was performed at the Image Analysis Laboratory of the National Cancer Institute (Frederick, MD).

### Mircoarray Analysis

RNA was isolated from two normal donor activated T cells at 0 or 6 h after OKT3 restimulation using Trizol (Invitrogen) and RNeasy mini-columns (Qiagen, Valencia, CA). Purified RNA was amplified using the Ovation Aminoallyl Amplification System (NuGEN, San Carlos, CA), labeled with Cy5 using the Cy5 Reactive Dye Pack (GE Healthcare, Piscataway, NJ), and cleaned up using Vivaspin columns (VivaScience AG, Hanover, Germany). Amplified RNA (2 μg) was hybridized to Hsbb 23K human spotted arrays (NIAID Mircoarray Research Facility) versus Cy3-labeled reference RNA pooled from six normal donor cycling T cells. Data was analyzed using GenePix and mAdb software.

### Immunoblotting

Cells were lysed in 1% NP-40 lysis buffer for 15 min on ice, then cleared by centrifugation. Protein concentration was determined by BCA assay (Pierce, Rockford, IL), and 20–30 μg total protein was separated by SDS-PAGE. Blots were probed with the following antibodies (Abs): anti-BIM (Stressgen, Ann Arbor, MI); anti-BAX, anti-cytochrome c (clone 7H8.2C12), anti-BCL-xL, anti-BCL-2, anti-MCL-1 (BD Pharmingen); anti-PUMA (Alexis); anti-β-actin (clone AC-15, Sigma). Bound Abs were detected using appropriate horseradish peroxidase-conjugated secondary Abs (Southern Biotech, Birmingham, AL) and ECL (Pierce).

### siRNA Transfections

Activated human PBL were transfected with 200 pmol of either specific small interfering RNA oligoribonucleotides (siRNA) or a non-specific (NS) control oligo (Invitrogen, Carlsbad, CA) using the Amaxa Nucleofection system (Amaxa, Koln, Germany). Assessment of knockdown efficiency and all subsequent assays were performed 4 days (human) post-transfection. siRNA sequences are available from Invitrogen (Stealth Select).

## Results and discussion

### TCR restimulation induces apoptosis signals independent of FAS

**Figure 1:**
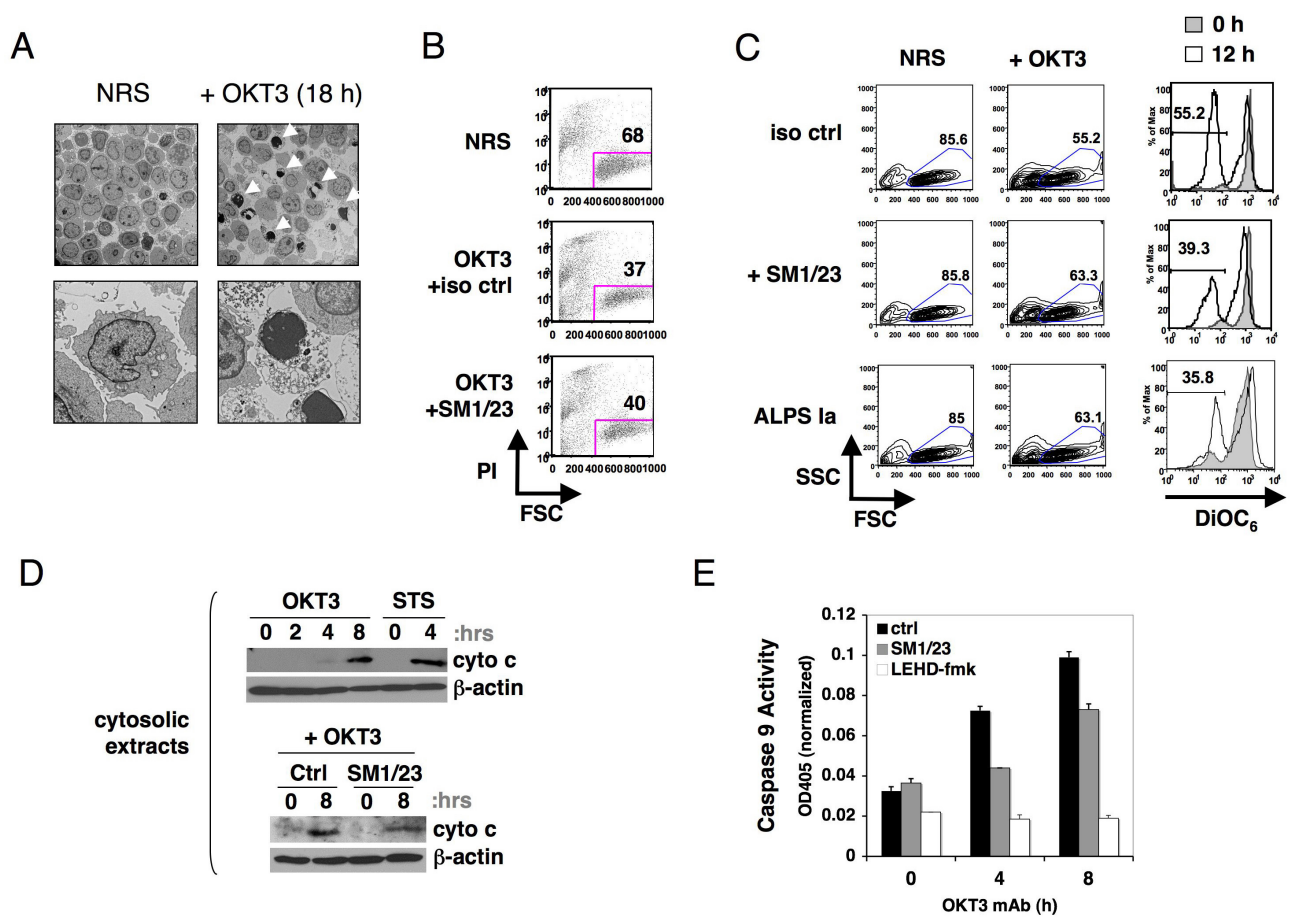
**TCR re-stimulation signals mitochondrial-dependent apoptosis independent of FAS**. (A) Electron micrographs (upper panels, 2500× magnification or lower panels, 10000×) of activated human PBL either not restimulated (NRS) or restimulated with OKT3 mAb for 18 h. Arrows indicate apoptotic cells. (B) Activated human T cells were untreated (NRS) or restimulated with OKT3 for 24 h in the presence of FAS blocking Ab (SM1/23) or isotype control Ab. Cells were stained with PI and analyzed by flow cytometry; gates indicate % viable cells. (C) Activated human T cells from a normal donor or ALPS 1a patient were untreated (NRS) or restimulated with OKT3 for 12 h in the presence of FAS blocking Ab (SM1/23) or isotype control Ab. Cells were stained with DiOC6 and analyzed by flow cytometry (right panels). Viable gates are shown at left, and the percentage of DiOC6 low cells are indicated in the histograms on the right. (D) Cytosolic extracts from activated human PBL were immunoblotted for the presence of cytochrome c following stimulation with OKT3 or staurosporine (STS) for the indicated timepoints, in the presence or absence of SM1/23 Ab. (E) Lysates prepared as described in (D) were incubated with the caspase 9 specific substrate LEHD-pNA for 2 h, and caspase 9 enzymatic activity was quantitated as OD at 405 nm minus background (OD405 at 5 min).

To examine TCR-induced death in human T cells, activated peripheral blood lymphocytes (PBL) from normal donors were restimulated with the anti-CD3 mAb OKT3 after cycling in IL-2 for 7–14 days. The majority of these cells are CD4^+ ^and CD8^+ ^T cells, with the latter generally more abundant in culture. Data was obtained for numerous human donors. We found that apoptosis was readily induced in restimulated T cells, marked by chromatin condensation and shrinkage (Figure 1A). This was followed by loss of membrane integrity due to secondary necrosis. Apoptosis was verified by PI exclusion; however, we noted that blocking FAS with an antagonistic Ab (SM1/23) provided only partial protection against TCR-induced death (Figure 1B). Flow cytometric analysis of restimulated T cells also confirmed cell shrinkage and loss of mitochondrial membrane potential, as indicated by decreased DiOC_6 _staining following 12 h of OKT3 treatment, signifying apoptosis (Figure 1C). Again, blocking Fas with an antagonistic Ab (SM1/23) only partially rescued this drop in mitochondrial membrane potential and cell viability. Remarkably, T cells from an ALPS Ia patient with a FAS death domain mutation also showed only a modest loss of mitochondrial membrane potential and viability (Figure 1C), suggesting a mitochondria-dependent apoptotic signal could proceed despite compromised FAS function. Similarly, cytochrome c released from mitochondria in response to OKT3 restimulation was only modestly decreased by FAS blockade (Figure 1D). We also tested caspase 9 activation, which occurs downstream of cytochrome c release and "apoptosome" formation. As expected, caspase 9 activation was only partially reduced in restimulated cells in the presence of FAS blocking Ab, but completely abrogated in the presence of the caspase 9 specific inhibitor LEHD-fmk (Figure 1E). In contrast, the SM1/23 Ab effectively blocked APO1.3 anti-Fas induced apoptosis, indicating that the cells were competent for FAS-mediated death (Additional File 1). Taken together, our data confirms that TCR-induced death relies in part on intrinsic mitochondrial signals triggered independently of FAS-FASL interactions.

### Role for BIM induction in TCR-induced death

**Figure 2:**
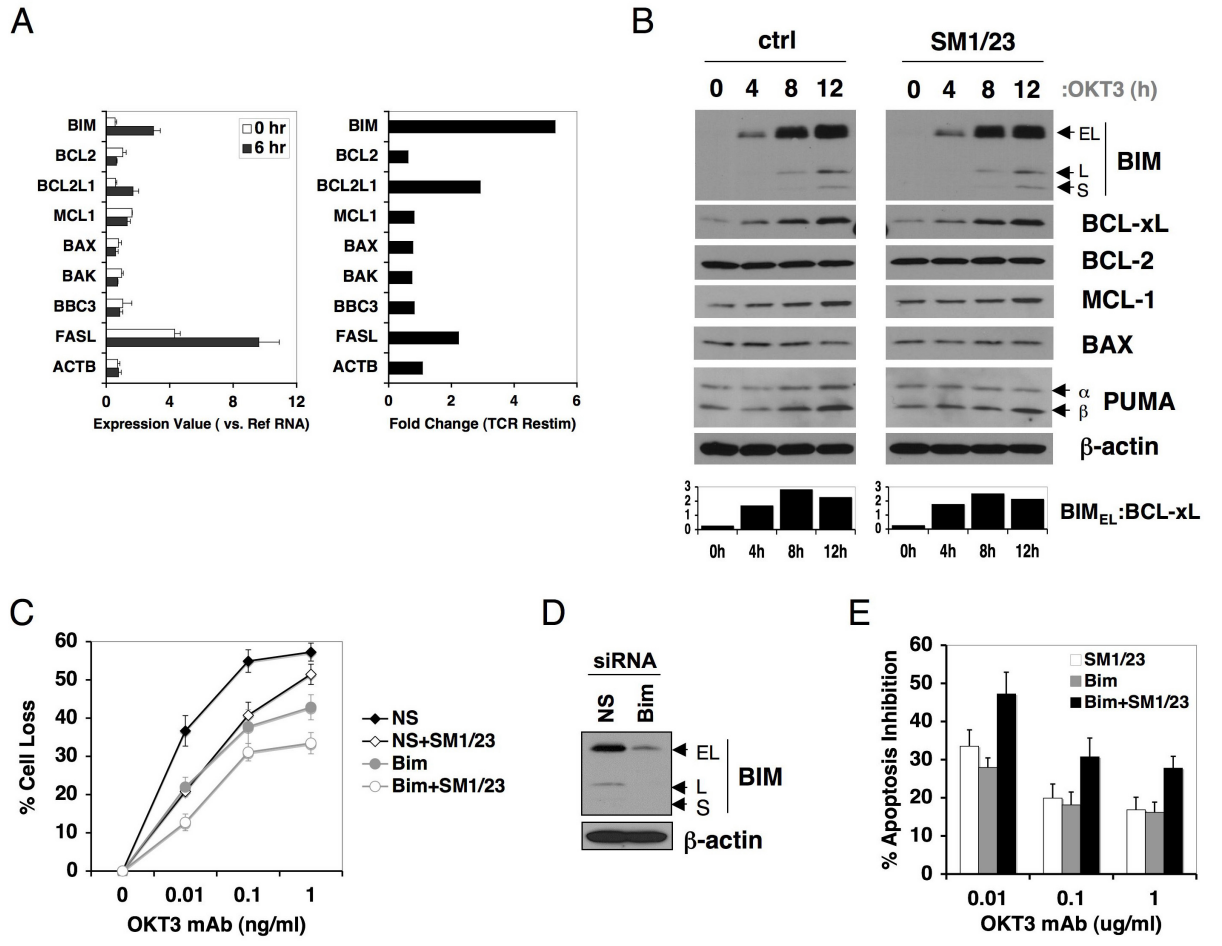
**Induction of BIM expression contributes to TCR-induced apoptosis**. (A) Mircoarray analysis of designated Bcl-2 family members was performed using RNA purified from activated human PBL either untreated (0 h) or stimulated with OKT3 for 6 h. Relative expression values normalized to reference RNA from normal human PBL are shown at left, fold change following TCR restimulation is quantitated at right. (B) Activated human PBL were stimulated with OKT3 for the indicated times, and whole cell lysates were prepared and immunoblotted for the proteins indicated on the right. All three isoforms of BIM (extra-long (EL), long (L), short (S)) were detected. Spot densitometry analysis of the ratio of BIM-EL to BCL-xL (normalized to β-actin loading control) is plotted below. (C) Activated human PBL were transfected with nonspecific (NS) or Bim-specific siRNA, rested 4 days, and then restimulated for 24 h with increasing doses of OKT3 in the presence or absence of SM1/23. Percent cell loss was calculated in triplicate by PI exclusion. Differences in apoptosis sensitivity (relative to NS alone) were statistically significant for each dose of OKT3 (p < 0.04), except for SM1/23 treated NS cells at 1 μg/ml. (D) Lysates from cells transfected in (C) were immunoblotted for BIM as in (B). β-actin serves as a loading control. (E) Average extent of TCR-induced apoptosis inhibition (relative to NS siRNA alone) is shown for each condition described in (D) for 6 different normal donor PBL tested.

Initial studies of AICD indicated that *de novo *transcription was required for the execution of apoptosis in response to T cell restimulation[17]. Since our data pointed toward a mitochondrial component, we surveyed expression of several pro- and anti-apoptotic BCL-2 family members using microarrays following TCR restimulation of activated human PBL for 6 h. As a positive control, we detected significant induction of FASL expression. Notably, we detected an even greater increase (> 5 fold) in BIM transcription in response to OKT3 stimulation (Figure 2A). Only BCL-xL was also increased with restimulation, whereas other BCL-2 family members remained largely unchanged or slightly decreased. The expression of all three BIM protein isoforms (extra long (EL), long (L), and short (S)) also increased substantially over time with OKT3 restimulation, whether Fas blockade was applied or not (Figure 2B). Although BCL-xL protein levels also increased, the ratio of BIM:BCL-xL expression rose substantially over time, suggesting heightened Bim expression represents a "tipping point" for overcoming the anti-apoptotic function of BCL-xL and related proteins in driving mitochondrial depolarization. PUMAβ levels also showed a minor increase (Figure 2B). Remarkably, the quick induction of BIM upon restimulation occurred in the presence of IL-2, which is required for TCR-induced death[6]. IL-2 signaling alone can destabilize BIM mRNA or promotes BIM protein degradation via Raf/ERK or phosphoinositide kinase 3 (PI-3K) signaling pathways [25-27]. However, our results suggest the TCR restimulation overrides this signal to allow for rapid BIM upregulation. These data are consistent with previous observations indicating BIM expression can be induced upon TCR triggering in human CTL clones, depending on the agonistic peptide used [28,29]. However, these studies did not establish whether loss of BIM expression had functional consequences for TCR-induced apoptosis sensitivity, or how this related to FAS-FASL signaling.

To definitively test whether BIM contributes to the TCR-induced apoptosis signal, we silenced BIM expression by RNA interference (RNAi) in activated PBL and restimulated them with OKT3 with or without FAS blockade. Knockdown of BIM expression significantly reduced the sensitivity of activated PBL to TCR-induced death (Figure 2C). Control immunoblots showed that BIM expression was silenced effectively in cells that received BIM-specific siRNA both before and after restimulation (Figure 2D, Additional File 2). As noted above, FAS blockade also partially rescued cells from death in these experiments, and had an additive protective effect when BIM expression was reduced (Figure 2C). The protective effects of BIM suppression and Fas blockade were noted in multiple human donors (Figure 2E). Knockdown of FAS associated death domain (FADD) rescued cells from TCR-induced death to a similar extent, further illustrating that death receptor signaling is only part of the apoptotic signal triggered by TCR restimulation (Additional File 3). In addition, knockdown of PUMA also provided some protection from TCR-induced death (Additional File 4), although this effect was variable in different donors tested. Collectively, our data definitively shows that intrinsic apoptosis mediators, particularly BIM, are required for optimal apoptosis triggered by TCR re-engagement separate from extrinsic FAS-induced apoptotic signals.

### Relative contribution of BIM in CD4^+ ^versus CD8^+ ^TCR-induced death

**Figure 3:**
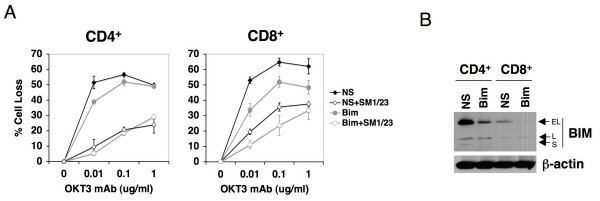
**Bim is important for TCR-induced apoptosis of CD8^+ ^T cells**. (A) CD4^+ ^or CD8^+ ^T cells purified from activated human PBL were transfected with NS or Bim-specific siRNA, rested 4 days, then restimulated with increasing doses of OKT3 in the presence or absence of SM1/23. Percent cell loss was calculated in triplicate by PI exclusion. Differences in apoptosis sensitivity were statistically significant for SM1/23 treated CD4^+ ^cells (NS and Bim) compared to NS cells alone (p < 0.007), except for SM1/23 treated NS cells at 1 μg/ml OKT3 (p < 0.07). Differences in apoptosis sensitivity for CD8^+ ^T cells (relative to NS alone) were all statistically significant (p < 0.05). (B) Lysates from cells transfected in (A) were immunoblotted for BIM. β-actin serves as a loading control.

We next tested whether BIM induction played a role in TCR-induced death of both CD4^+ ^and CD8^+ ^T cells. Purified CD4^+ ^and CD8^+ ^T cells sorted from activated PBL were transfected with NS or BIM-specific siRNA and tested for sensitivity to OKT3-induced death. Whereas Fas blockade alone substantially rescued the apoptosis of purified CD4^+ ^T cells, knockdown of Bim expression had little effect (Figure 3A). Conversely, CD8^+ ^T cells relied on both FAS and BIM for TCR-induced apoptosis signaling. Although BIM expression was consistently higher in CD4^+ ^T cells compared to CD8^+ ^T cells from multiple donors (Figure 3B), BIM induction from steady state levels was as good or better in CD8^+ ^T cells upon restimulation (Additional Files 2 &5). We cannot rule out that residual BIM expression in CD4^+ ^T cells following BIM siRNA transfection contributed to the Fas-independent of apoptosis observed. However, other experiments revealed that BIM knockdown using the same siRNA provided greater protection from IL-2 withdrawal apoptosis in CD4^+ ^T cells (Additional File 6), suggesting BIM levels could be sufficiently depleted to hinder BIM-dependent death. Collectively, the data suggests that human CD8^+ ^T cells rely on BIM more extensively for TCR-induced deletion than CD4^+ ^T cells, which are largely dependent on FAS signaling. This idea agrees with landmark studies that implicated FAS in TCR-induced apoptosis, which focused primarily on CD4^+ ^T cell lines or clones from humans or mice [15-18]. Moreover, our data potentially explain new studies suggesting BIM drives Ag-specific CD8^+ ^T cell deletion in establishing peripheral tolerance in both mice and humans [30,31].

### Bim and Fas cooperate in TCR-induced apoptosis of murine T cells

**Figure 4:**
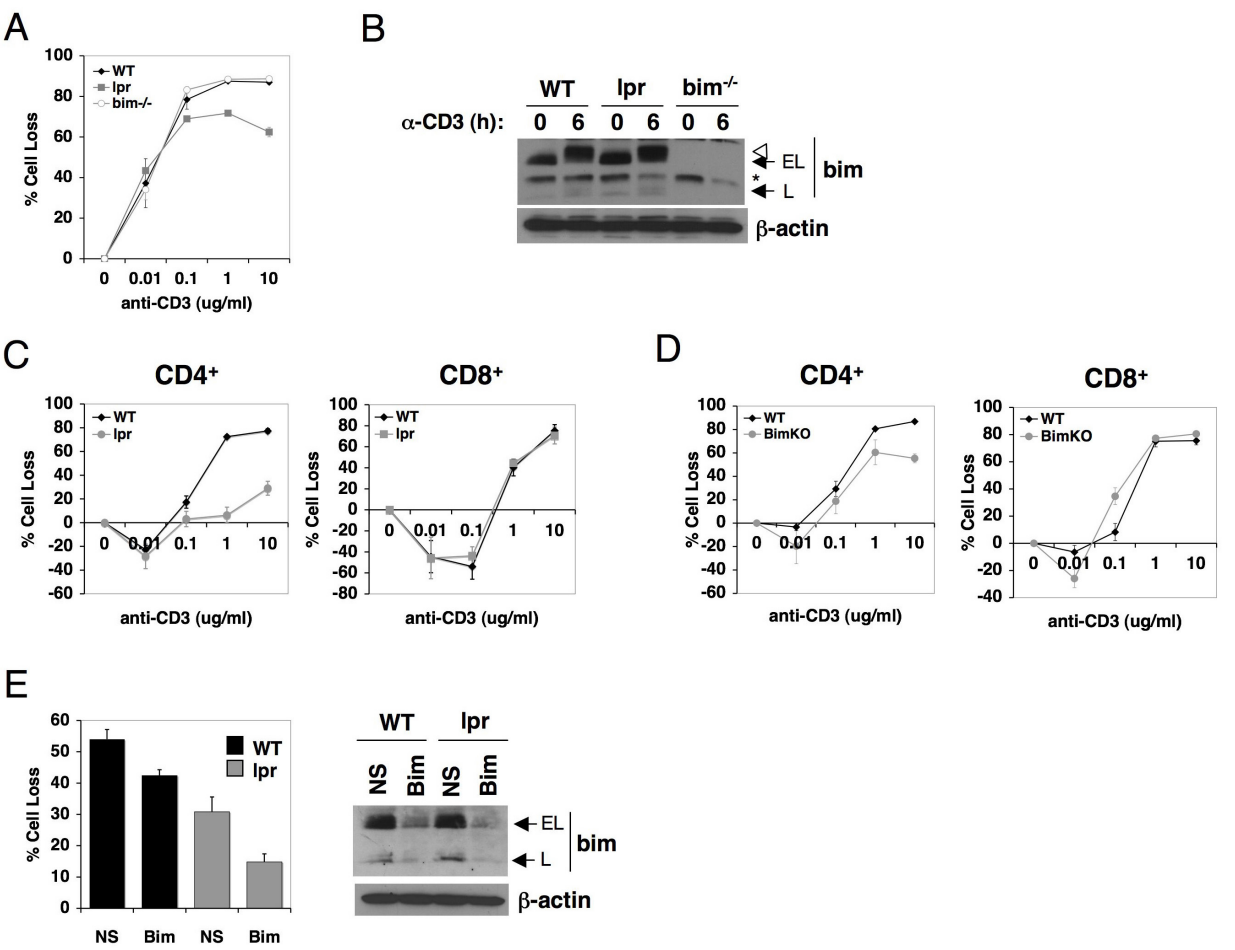
**Fas and Bim cooperate in driving TCR-induced apoptosis of murine T cells**. (A) Activated splenic T cells from wild-type (WT), *lpr*, or *bim*^-/- ^mice were restimulated with platebound anti-CD3 for 24 h. Percent cell loss was calculated in triplicate by PI exclusion. (B) Lysates from splenic T cells from the indicated genetic backgrounds left untreated or restimulated with platebound anti-CD3 for 6 h were immunoblotted for Bim isoform expression. β-actin serves as a loading control; asterisk indicates non-specific band. (C, D) CD4^+ ^and CD8^+ ^T cells were purified from activated WT, *lpr*, or *bim*^-/- ^splenocytes and restimulated with platebound anti-CD3 for 24 h. Percent cell loss was calculated in triplicate by PI exclusion. (E) Splenic T cells from WT or *lpr *mice were stimulated for 48 h with platebound anti-CD3/anti-CD28, washed, and transfected with NS or Bim-specific siRNA. Three days post-transfection, cells were restimulated with 100 ng/ml platebound anti-CD3; percent cell loss was calculated in triplicate by PI exclusion. Differences in apoptosis sensitivity (relative to NS-treated WT cells) were statistically significant (p < 0.04). Lysates made from cells three days post-transfection were assessed for Bim knockdown by immunoblotting, right.

In light of our findings in human T cells, we re-examined TCR-induced death in murine T cells. Surprisingly, we observed that activated splenic T cells from Fas-deficient *lpr *mice showed only minor resistance to anti-CD3-induced death induced by restimulation, whereas *bim *knockout mice showed no difference in sensitivity compared to WT cells (Figure 4A). We also tested for Bim induction in restimulated WT and *lpr *T cells in the presence of IL-2. Consistent with data in human T cells, activated mouse T cells (WT or *lpr*) showed a clear increase in BimEL expression after 6 hours of restimulation (Figure 4B). We also detected a change in the migration of BimEL and BimL isoforms, suggesting post-translational modifications may affect of bim function in mice, perhaps via phosphorylation.

Next, we reasoned that differences in apoptosis sensitivity caused by loss of Fas or Bim may differ in CD4^+ ^and CD8^+ ^T cell cultures, as noted in for human T cells. Therefore, we assayed TCR-induced apoptosis sensitivity in purified CD4^+ ^and CD8^+ ^T cells from WT, *lpr*, and *bim*^-/- ^mice. As expected from previous reports, CD4^+ ^*lpr *cells showed a profound defect in restimulation-induced death (Figure 4C). This concurred with our results in human CD4^+ ^T cells using Fas blocking Ab (Figure 3A), indicating Fas is necessary for CD4^+ ^T cell restimulation apoptosis. In contrast, there were no differences in CD8^+ ^T cell death between restimulated WT and *lpr *cells, explaining the cumulatively minor rescue of TCR-induced death in total splenic T cells when Fas is absent. Furthermore, genetic ablation of *bim *had little protective effect for activated CD4^+ ^T cells upon TCR restimulation, and no discernible effect on apoptosis in CD8^+ ^T cells (Figure 4D).

We hypothesized that loss of Bim from development, through germline gene ablation, may permit T cells to "compensate" accordingly via enhanced expression or function of pro-apoptotic molecules. Therefore, we acutely silenced Bim using RNAi in activated WT and *lpr *T cells. Knockdown of Bim significantly protected activated WT and *lpr *T cells from apoptosis induced by 100 ng/ml anti-CD3 stimulation (Figure 4E, left panel), demonstrating that Bim can play a prominent role in this apoptosis pathway. This effect was also noted in purified CD4^+ ^and CD8^+ ^T cell populations (data not shown), even though loss of Fas alone reduced sensitivity only in CD4^+ ^T cells again as expected. Control blots showed that Bim siRNA effectively suppressed Bim protein expression in both WT and *lpr *T cells (Figure 4E, right panel). This protective effect was less pronounced at higher doses of anti-CD3 stimulation (data not shown), suggesting stronger restimulation may override Bim siRNA effects and/or trigger alternative death effector pathways. Thus, our data suggests that Fas or Bim may partially compensate for the loss of one or the other from development in murine T cells during development.

### Relative BIM expression correlates with sensitivity to TCR-induced death in ALPS patients

**Figure 5:**
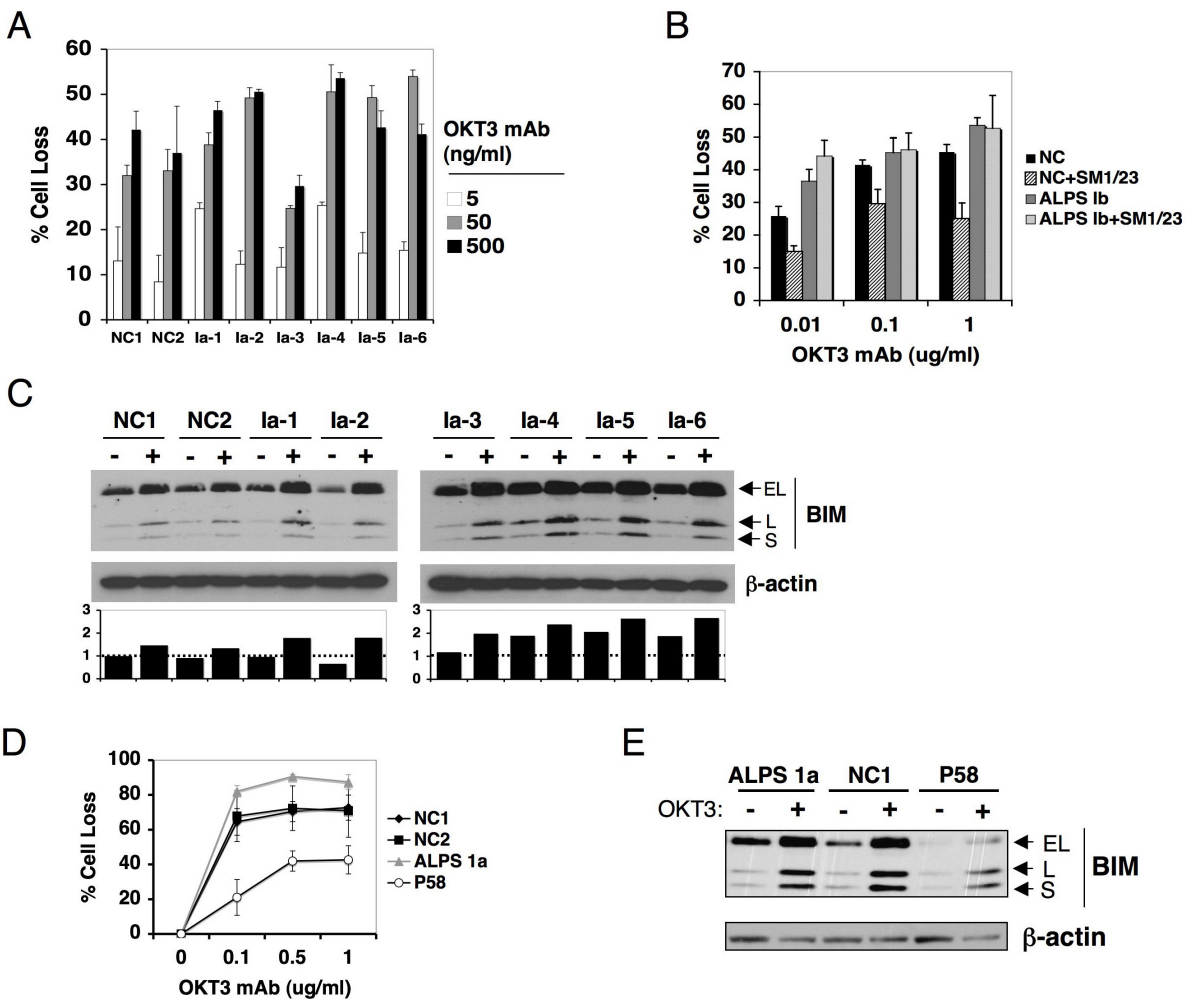
**Suppression of Bim expression in ALPS type IV patient causes resistance to TCR-induced death**. (A) Activated human PBL from normal control donors (NC1, NC2), or 6 ALPS type Ia patients were restimulated with increasing doses of OKT3 for 24 h. Percent cell loss was calculated in triplicate by PI exclusion. (B) Activated human PBL from an ALPS type Ib patient or a normal control donor (NC) were treated as in (A). Percent cell loss was calculated in triplicate by PI exclusion. (C) Activated human PBL from normal control donors (NC1, NC2), or 6 ALPS type Ia patients restimulated with 100 ng/ml OKT3 for 0 (-) or 8 h (+), lysed and immunoblotted for BIM. β-actin serves as a loading control. Spot densitometry analysis of the ratio of BIM (EL isoform) to β-actin (normalized to NC1 untreated, dashed line) is plotted below. (D) Activated human PBL from normal control donors (NC1, NC2), an ALPS type Ia patient, and an ALPS Type IV patient (P58) were restimulated with OKT3 for 24 h. Percent cell loss was calculated in triplicate by PI exclusion. Differences in apoptosis sensitivity (relative to NC1 or NC2) were statistically significant (p < 0.01). (E) Activated human PBL as in (D) were restimulated with 100 ng/ml OKT3 for 0 (-) or 8 h (+), lysed and immunoblotted for BIM. β-actin serves as a loading control.

Based on our aforementioned results, we revisited TCR restimulation-induced apoptosis in PBL derived from several ALPS patients. Similar to controls, PBL cultures from ALPS Ia patients were primarily comprised of CD8^+ ^T cells (data not shown). Surprisingly, we found that PBL from several ALPS Ia patients displayed normal or slightly more death in response to OKT3 titration compared to normal controls, despite impaired apoptosis upon direct Fas crosslinking. (Figure 5A, Additional File 7). Similarly, T cells derived from an ALPS Ib patient harboring a dominant interfering mutation in *FASL *[32] were also killed effectively upon TCR restimulation (Figure 5B). Consistent with defective FASL function, TCR-induced apoptosis was unaffected by Fas blockade. These results exposed a glaring contradiction in the concept that FAS mediates most or all TCR-induced death.

We next assessed the relative expression of BIM before and after restimulation of PBL in ALPS Ia patients. In general, we noted higher BIM protein expression in restimulated ALPS Ia T cells relative to controls (Figure 5C). In 4/6 ALPS Ia patients, steady-state BIM expression was also elevated relative to controls. Using spot densitometry, we estimated that ALPS Ia T cells had between 30–80% more BIM protein than normal controls both before and after TCR ligation (Figure 5C, bottom panel). BIM siRNA treatment did not result in a significantly greater rescue of TCR-induced death in ALPS Ia cells compared to normal controls (data not shown), perhaps due to incomplete depletion of BIM or compensation by other mediators (e.g. PUMA). Nevertheless, elevated BIM levels in cycling T cells with defective FAS function may suggest that these T cells are "primed" for apoptotic deletion through a compensatory increase in BIM expression.

Finally, we tested TCR-induced apoptosis in T cells derived from an ALPS Type IV patient (P58) with a gain-of-function, germline NRAS mutation that constitutively activates ERK and suppresses BIM expression. We recently demonstrated that P58 T cells are resistant to apoptosis induced by IL-2 withdrawal due to BIM suppression[12]. Remarkably, P58 T cells displayed partial resistance to TCR-induced death when compared to normal donor and ALPS Ia cells, despite comparable expression of FAS on the cell surface (Figure 5D, Additional File 8). Moreover, BIM expression was attenuated in P58 T cells and could not be rescued by TCR restimulation (Figure 5E), providing stronger evidence that BIM serves a physiologically relevant role in the restimulation apoptosis pathway, especially for CD8^+ ^T cell homeostasis. Moreover, our data implies that relative BIM expression may represent an important determinant of TCR-induced apoptosis sensitivity, independently of FAS. However, we concede that NRAS/ERK dysregulation in P58 could alter TCR-induced death through BIM-independent mechanisms as well. Indeed, pharmacological ERK inhibitors actually provided a small but reproducible rescue of TCR-induced death in both normal and P58 T cells.

The physiological function of Bim was originally revealed from characterization of Bim-deficient mice, from which T cells were profoundly resistant to lymphokine withdrawal death[8]. The pro-apoptotic function of Bim also enforces immune tolerance through thymocyte negative selection, CD8^+ ^T cell cross tolerance, and the regulation of antigen presenting cells including B cells and dendritic cells[23,33-35]. Here we demonstrate that BIM also plays a significant role in TCR-induced death of activated human T cells, working in tandem with FAS signaling as a separate signal to kill T cells. This provides a new mechanism besides the cleavage of BID for an extrinsic signal to activate the intrinsic mitochondrial death program. This paradigm may be distinct from Bim-dependent "activated T cell death" described by Hildeman et al. in mice challenged with a single dose of superantigen [36], which may be interpreted as predominantly cytokine withdrawal apoptosis, not restimulation-induced death with repeated Ag dosing. On the other hand, the marked accumulation and persistence of Bim-deficient murine CD8^+ ^T cells in chronic viral infection models could be connected to failed deletion in response to repeated TCR stimulation [37,38].

Our results show that direct signals from the TCR program T cells to die through Bim, which is fundamentally different from the secretion of death cytokines such as FasL that engage external death receptors. This has some interesting implications. First, it may be advantageous in conditions where Fas may not be effective. For example, Bim has a greater influence in CD8^+ ^T cells that can utilize FasL:Fas as a calcium-independent cytolytic mechanism against infected target cells and therefore may be inured to its lethal effects. Second, the direct molecular connection inside the cell may make the Bim pathway more efficient. Careful investigation of the temporal effects of killing after TCR engagement may reveal differences between Fas and Bim effectiveness. Third, as Bim expression is extensively regulated post-translationally, the fact that translation inhibitors only partially block TCR-induced death could indicate there is a direct death pathway entrained to TCR restimulation that does not require new protein synthesis [17]. Finally, pro-apoptotic mediators like Bim or Puma acting at the convergence of TCR and CWA may help to restrain these pathways at a focal point for tight control of those T cells escape death and emerge as memory T cells.

Recently, three groups reported that loss of both Bim and Fas in mice results in massive lymphadenopathy/splenomegaly, early onset of SLE-like autoimmune manifestations, and even greater accumulation of antigen-specific CD8^+ ^T cells upon chronic viral infection [39-41]. These experiments reprised earlier work that obtained very similar results when transgenic Bcl-2 overexpressing mice were crossed onto an *lpr *background [42,43]. However, their general conclusions still emphasized the traditional model, reiterated in an accompanying review, that Fas and Bim control T cell homeostasis through two distinct pathways: restimulation-driven versus IL-2 withdrawal-induced apoptosis, respectively[5,7,19]. Our study illustrates that death of activated T cells via Fas or Bim are not mutually exclusive pathways, as both can operate in IL-2 dependent TCR-induced apoptosis. During infections this combination of potent extrinsic and intrinsic signals may act to ensure rapid and efficient killing of hyper-responsive or cross-reactive autoimmune T cells upon repeated antigen encounter, thus preventing immunotoxicity and maintaining peripheral tolerance. Another intriguing possibility relates to the potential of Bim and Fas to partially compensate for one another in driving TCR-induced apoptosis. This applies to situations where either gene function is lost from development, such as in *lpr *or *bim*^-/- ^mice, and may explain why only acute knockdown of Bim resulted in significant reduction of TCR-induced apoptosis in murine T cells *in vitro*. The idea that Bim participates in ensuring T cell homeostasis both during and after effector T cell responses may also explain why Bcl-2 Tg *lpr *mice described years ago have strikingly worse lymphocyte accumulation compared to either Bcl-2 Tg or *lpr *mice alone[43]. Our results provide a new interpretation of the mouse studies by revealing that the infection-induced derangement of T cell homeostasis caused by Bim-deficiency could be accounted for by an impairment of both intrinsic and extrinsic apoptosis. It is also notable that ALPS patients show wide variability in conventional CD3^+ ^T cell numbers, with a substantial fraction showing no increases. By contrast, the fraction and absolute number of "double negative" (CD4^-^CD8^-^) α/β T cells are invariably elevated [44]. This may reflect that alternative effectors such as BIM could preserve equipoise in the conventional T cell compartment.

In humans, our data are consistent with previous studies suggesting that TCR-induced death involves multiple effector molecules, and clearly includes components other than FAS or BIM that remain to be elucidated[22]. We have previously noted a role for tumor necrosis factor-alpha (TNF-α) in this process for murine CD8^+ ^T cells[21]; however, blockade of this pathway in human T cells had little demonstrable effect (data not shown), which requires further exploration. A recent paper from Mateo et al. implicated perforin and cytotoxic granules in the execution of TCR-induced death, particularly for ALPS Ia patient cells[45]. Other inputs implicated in control of AICD sensitivity, including NF-κB regulation through HPK-1 or generation of reactive oxygen species (ROS), likely relate more to the regulation of FasL or Bim expression[46,47]. However, we are studying patients with impaired TCR-induced apoptosis despite normal induction of FASL and BIM, and normal apoptosis triggered by FAS ligation or IL-2 deprivation (A.L. Snow, unpublished data). Insights gleaned from such patients may further advance our understanding of the biochemical complexity and physiological relevance of apoptosis in different immune cell compartments. Nevertheless, our current findings further elucidate FAS-independent signals for the restimulation-induced death of activated T cells via BIM induction.

## Conclusion

Although Fas-FasL signaling is often considered synonymous with TCR restimulation-induced death, the data provided herein show it has a quantitatively lesser role than previously acknowledged, and support a critical role for BIM induction in the execution of antigen-driven "extrinsic" apoptosis. Increased BIM expression following TCR restimulation, even with a surfeit of IL-2, works in parallel to FAS signaling in driving mitochondrial depolarization, caspase 9 activation, and eventual apoptosis. Like FAS blockade, suppression of BIM induction via RNAi or increased NRAS activity in an ALPS variant patient results in partial resistance to TCR-induced death. These data build upon previous work from Sandalova and colleagues by demonstrating that BIM is indispensable for maximum sensitivity to restimulation-induced apoptosis of human T cells. More importantly, our findings revise previous models in showing that FAS and BIM both participate in eliminating activated T cells through this IL-2 dependent, propriocidal death pathway.

## Abbreviations

ALPS: Autoimmune lymphoproliferative syndrome; FasL: Fas ligand; PBL: Peripheral blood lymphocytes; PI: Propidium iodide.

## Competing interests

The authors declare that they have no competing interests.

## Authors' contributions

ALS and JBO designed and performed all experiments. ALS wrote the manuscript. LZ helped with activation and analysis of ALPS Type Ib cells. JKD. cared for ALPS patients and facilitated sample collection and distribution. MJL and TAF helped direct the research and contributed to final revisions.

## Reviewers' comments

Reviewer #1, Snow et al.:

Dr. Wendy Davidson, University of Maryland School of Medicine

**Editor: **Dr. David Scott, University of Maryland School of Medicine

This manuscript examines the role of BIM in TCR restimulation-induced death in human T cells from healthy individuals and patients with ALPS. The authors show that restimulation of normal unfractionated T cell blasts results in significant upregulation of BIM expression and that reactivation-induced cell death (AICD) is reduced when FAS/FASL interactions are blocked with antagonist Ab or BIM expression is decreased by siRNA. Blockade of both pathways appeared to have additive effects suggesting that both BIM- and FAS-mediated death pathways contribute to AICD. Further analysis of T cell subsets showed that although CD4^+ ^T cell blasts express higher levels of BIM than CD8^+ ^T cell blasts prior to and after restimulation, BIM-mediated death appears to be restricted to CD8^+ ^T cells. Combined, the data on subsets suggest that AICD in CD4^+ ^T cells is predominantly FAS-mediated whereas CD8^+ ^T cells are susceptible to death induced by FAS and BIM. Additional studies with unfractionated T cell blasts from ALPS patients deficient in FAS or FASL indicated sensitivity to FAS-independent AICD, increased basal levels of BIM and increased expression of BIM post restimulation. However, attempts to block AICD using BIM siRNA were unsuccessful. As an alternative indirect approach to determine the contribution of BIM to AICD in the context of ALPS, the investigators used T cells from an ALPS type IV patient with a germline NRAS mutation resulting in constitutive activation of ERK and suppression of BIM expression. Unfractionated T cell blasts from this patient were efficiently killed by anti-FAS mAb but were relatively insensitive to TCR-induced death, suggesting that BIM might contribute to AICD.

The findings with normal human CD8^+ ^T cells are novel and consistent with recent studies in mice indicating the importance of both the BIM and FAS cell death pathways in regulating CD8^+ ^T cell homeostasis. The data relating to the role of BIM in T cell death in ALPS patients are less compelling because of the difficulties in efficiently downregulating BIM in T cell blasts from ALPS type I patients. Further, in the ALPS type IV patient the authors assume, but do not prove, that the only way that dysregulated expression of NRAS protects T cells from restimulation-induced death is through down regulation of BIM.

Critique:

Major points:

1. The key data shown in Figs. 2C and 3A appear to be from a single experiment utilizing cells from one individual. The investigators should indicate how reproducible the studies were and whether there was significant variation in susceptibility to BIM-mediated killing among T cells from different individuals.

### Author Response

*The reviewer poses an important question. Although some variation was noted in cells from different donors, the experiments described were very reproducible in multiple replicate experiments, utilizing several human donors. In order to clarify this point, Figure *2E* has been added to summarize the extent of TCR-induced apoptosis inhibition, relative to NS siRNA-transfected cells, for 6 different human donors tested.*

2. In Fig. 2D, the authors show that BIM expression is significantly reduced in T cell blasts after transfection with BIM siRNA. The authors do not specify whether the cell lysates came from blasts 4 days post transfection or from restimulated transfected blasts. Similar data shown in Additional File 2 indicate that western blots were performed prior to restimulation. It is imperative that the authors demonstrate that restimulation-induced upregulation of BIM is prevented in the BIM siRNA transfected blasts. Otherwise, the data cannot be interpreted.

### Author Response

*We agree with the reviewer's critique, as blots shown represent cells lysed 4 days post-transfection of siRNA without restimulation. To address this point, the new Additional File *2* now demonstrates that Bim siRNA effectively suppresses BIM expression/induction in both resting and restimulated T cells.*

3. Data in Fig. 3A suggest that BIM either does not contribute to restimulation-induced death in CD4^+ ^T cell blasts or that the BIM death pathway is far less efficient than the FAS death pathway in inducing apoptosis in this population. Studies with Bim KO mice may help to distinguish between these two possibilities, assuming that murine CD4^+ ^T cells reliably model human CD4^+ ^T cells in this regard. Decreased sensitivity of BIM knockdown CD4^+ ^blasts to IL-2 withdrawal-induced death would provide additional evidence that BIM levels are sufficiently depleted to impair BIM-dependent death.

### Author Response

*Following the reviewer's suggestion, we have added a new figure detailing several experiments performed with mouse T cells from Bim KO as well as lpr mice (new Figure *4*). These data support the conclusion that CD4^+ ^T cells are much less dependent on Bim compared to Fas for TCR-induced death, although we found that CD8^+ ^T cells were equally sensitive to the blockade of either pathway. On the other hand, acute knockdown of Bim expression in mouse T cells rescued a substantial proportion of WT T cells, and almost all lpr T cells, from TCR-induced apoptosis. We hypothesize that Bim KO cells maintain sensitivity to TCR-induced death through a compensatory effector molecule (Fas or otherwise), such that the acute knockdown in WT cells does not allow sufficient time or the physiological context for a hypothetical compensatory mechanism to have a significant effect.*

*We also provide new data (Additional File *6*) showing that our RNAi approach provides sufficient depletion of BIM to impair BIM-dependent, IL-2 withdrawal apoptosis in CD4^+ ^T cells. In fact, the extent of this protection was more pronounced for CD4^+ ^T cells compared to CD8^+ ^T cells. This contrast suggests the more prominent role of BIM in human CD8^+ ^T cell deletion, supported by our data in Figure *3*, is specific to TCR restimulation-induced death.*

In Fig. 2C, the data suggest that the FAS and BIM death pathways are equally effective in killing blasts enriched for CD8^+ ^cells. However, in Fig. 3A purified CD8^+ ^blasts appear to be more sensitive to FAS-mediated death. This difference deserves comment. The author's should also indicate whether the greater sensitivity of CD8^+ ^blasts to FAS-mediated death is a consistent finding in healthy humans. As discussed above for the unfractionated blast populations, data showing that siRNA blocks the restimulation-induced increase in BIM expression in the purified CD4^+ ^and CD8^+ ^are essential.

### Author Response

*In general, we cannot definitively say that purified CD8^+ ^T cells are more sensitive to the Fas-FasL component of TCR-induced death than unfractionated PBL. The data shown in Figure *3A* are representative of several experiments, although some variability in the extent of rescue by the Fas antagonist antibody SM1/23 was noted in both purified CD8^+ ^T cells and PBL. As noted in the text, the majority of T cells found in normal donor PBL (roughly 55–80%) were CD8^+ ^T cells following a week of cycling in IL-2. As mentioned above, Additional File *2* demonstrates that Bim-specific siRNA blocks the restimulation-induced increase in BIM expression in purified T cell subsets.*

4. Although T cells from ALPS patients with defects in FAS signaling are clearly susceptible to AICD and exhibit increased expression of BIM after restimulation, no conclusions can be drawn regarding the contribution of BIM in FAS-independent apoptosis in CD4^+ ^or CD8^+ ^T cells since the authors were unable to block BIM expression. While the ALPS type IV patient, P58, with reduced BIM levels and decreased sensitivity to AICD provides additional preliminary evidence for a role for BIM in T cell homeostasis, the authors should address the possibility that the enhanced cell survival associated with NRAS dysregulation may result from effects other than, or in addition to, BIM downregulation.

In Fig. 4D, the investigators show that P58 blasts are highly sensitive to anti-FAS-induced apoptosis but do not establish whether FAS-mediated AICD is intact. Ideally, the AICD experiments should be repeated with isolated P58 CD4^+ ^and CD8^+ ^blasts in the presence or absence of antagonist anti-FAS Ab to determine the level of FAS-mediated apoptosis in each population. If P58 CD4^+ ^blasts are sensitive to AICD and death is significantly inhibited by FAS blockade, the investigators will have additional evidence that BIM contributes minimally to AICD in CD4^+ ^T cells.

### Author Response

We appreciate the authors' interpretation. Accordingly, we now provide additional commentary in the Discussion to point out that the gain-of-function NRAS mutation in P58 T cells could be altering TCR-induced death signaling through additional unidentified BIM-independent pathways. Although purified CD4^+ ^and CD8^+ ^T cells were not tested, TCR-induced death in P58 PBL could be partially rescued by Fas blockade to a similar extent as normal donor T cells, suggesting FasL function is intact. We also concur that enhanced BIM expression in ALPS Ia patients is suggestive, but not definitive, evidence for BIM-dependent AICD short of better knockdown experiments.

Minor Points:

1. In their discussion (page 14, line 10), the authors overstate the differences between their interpretation of how FAS and BIM may work to regulate T cell homeostasis and the conclusions of three other groups studying mice deficient in Bim and functional Fas.

### Authors Response

We respectfully disagree with the author's opinion in this case, as we feel it is imperative to highlight the novelty of our findings in relation to the three cited articles pertaining to Bim-deficient lpr mice. The "preview" article published alongside those articles in Immunity (see references in the manuscript) maintains that Fas controls antigen-stimulated or "active" death, and cytokine withdrawal death governed by Bim and its interactions with other Bcl-2 family members controls antigen depletion or "passive" death in a mutually exclusive manner. Actually our data indicate that both of these contentions are oversimplified. Ample data indicates that Fas is not the only mediator of TCR-stimulated death of activated cells illustrated in all of the experiments in this paper by the fact that substantial residual death evident even with combined Fas/Bim blockade. Moreover, our data demonstrate that Fas and Bim can both contribute to apoptosis caused by TCR restimulation of activated T cells. This provides a different molecular schema for interpreting the synergistic increase in lymphadenopathy and autoimmunity they reported resulting from combined genetic lesions of Fas and Bim. It is clear from those papers and the overview written by Green that the interpretation is confined to a model that has existed in the field for over a decade, viz Lenardo, J. Exp. Med. 183: 1071, 1996. Our new findings reveal a functional connection between the TCR-induced pathway and Bim that was previously hinted at in the literature (Sandalova et al., Hum Immunol. 2006 Dec;67(12):958–65) and not considered in the 2008 Immunity papers. This sheds new light on the described phenotype of these mouse experiments, particularly with regard to excessive CD8^+ ^T cell lymphoproliferation, that was not considered.

2. Since the difference in susceptibility of CD4^+ ^and CD8^+ ^T cells to BIM-mediated AICD is a novel observation, the authors should discuss this data more extensively.

3. Information on the source of the SM1/23 mAb and a reference for the cell death assay need to be included in the Methods section.

4. In Fig. 4B, the axis label for OKT3 concentration should read μg/ml. On page 7 there is are typos in the spelling of microarray. Data is plural.

### Author Response

We have elaborated on our discussion of differences in BIM-driven TCR-induced death of CD4^+ ^and CD8^+ ^T cells as recommended by the reviewer. We have also added two very recent references that demonstrate a prominent role for BIM in Ag-specific peripheral deletion of CD8^+ ^T cells in mice and humans, in agreement with our in vitro data. The information requested is now provided in the text, and typos have been corrected.

Reviewer #2, Snow et al.:

Dr. Mark Williams, University of Maryland School of Medicine

Editor: Dr. Neil Greenspan, Case Western Reserve University

Recent papers suggest that the role of Bim in classic TCR-induced AICD is not well defined. This paper addresses this question.

The data in Figure 2 suggest that Bim plays a role in AICD in the T cells from normal controls, but the data from the ALPS patients in Figure 4 do not allow such an interpretation. The P58 cells could be resistant to AICD for many other reasons besides a lack of Bim. For example FasL levels are not examined or TCR signals needed to sensitize to AICD may be deficient. The Bim siRNA data in ALPS patients also should be shown (page 13).

### Authors Response

*We concede that because we were unable to knockdown BIM expression adequately in ALPS Ia patient cells, data shown in Figure *5C* do not conclusively show that BIM plays a more prominent role in TCR-induced death of ALPS Ia patient cells. However, elevated BIM expression noted in several ALPS Ia patients strongly suggests that other apoptosis effector molecules, including BIM, may be compensating for the loss of functional Fas in maintaining sensitivity to TCR-induced death. The striking sensitivity of ALPS cells to TCR-induced death is a surprising effect that we believe has been unequivocally demonstrated. Indeed, a similar interpretation may be derived from our new data in Bim KO and lpr T cells (see new Figure *4*). Please note our response to Reviewer 1 with regard to interpreting data from P58 cells.*

Statistical analysis is needed for all graphs (the word "significantly" is used throughout the text) especially in Figure 3 to establish that Bim knockdown gives additional protection above Fas blockade.

### Authors Response

We point out that statistical analysis (paired T tests) was applied to all of the graphs shown throughout the paper, as noted in the Figure Legends (p values are included). This statistical analysis is now mentioned in the Methods section for clarification. The word "significantly" was applied in the manuscript with these statistical analyses in mind, referring only to differences with p < 0.05.

Overall, the data support a role for Bim in AICD, albeit not as strongly as the authors suggest. More studies in which Bim expression is manipulated are necessary to support such a conclusion.

### Authors Response

*Currently, direct silencing of BIM using siRNA is our most effective tool for manipulating BIM expression in primary human T cells. Unfortunately, ectopic expression of BIM in primary cells is more problematic, as cell viability is poor due to both BIM overexpression and increased cell loss during electroporation of an expression plasmid. We hope that the inclusion of new data in Figure *2E* and Additional File *2* strengthens our conclusion that BIM siRNA effectively suppresses BIM expression during restimulation, translating to AICD resistance in multiple human donors tested. Furthermore, the effect of acute Bim knockdown in WT and lpr mouse T cells resembles our data in human T cells and supports our general hypothesis further.*

Reviewer #3, Snow et al.:

Editor: Laurence C. Eisenlohr, Thomas Jefferson University

Comments:

1. In reference to the term "propriocidal cell death": I'm familiar with "proprioception" but not "propriocidal".

### Author Response

We employ the term "propriocidal", based on the Latin prefix ("proprio" = "of his own accord"), to refer to TCR-induced apoptosis as a self-regulatory form of T cell death. This has been introduced in the literature on this topic in the past: Nature 353: 858–861, 1991, Eur. J. Immunol., 23: 1552–1560, 1993. Although this term may be somewhat recondite, it is interesting that the Editor draws the intended analogy. Just as the proprioceptive system of inhibitory neurons provides negative feedback to control motor movements, we theorize that the negative feedback of propriocidal T cell death can govern the intensity of an immune response to prevailing conditions of antigen and IL-2. To avoid confusion we will clarify this term in the Introduction and Discussion.

2. In reference to first sentence of "Conclusions" section of Abstract: This sentence is a speed bump for me. This is the first mention of IL-2 but the implication is that you see BIM upregulation under both conditions – restimulation and IL-2 withdrawal. Is the IL-2/BIM connection already published?

### Author Response

As explained in the Introduction, the connection between IL-2 withdrawal and BIM upregulation is well established in the literature. However, we emphasize our novel finding that robust BIM upregulation in the presence of IL-2 upon TCR restimulation suggests a different signal emanating from the TCR can override BIM destabilization normally conferred by IL-2R signaling.

3. In reference to Introduction (pg 4, paragraph 2): This sentence seems a little off point. If I understand correctly, it is illustrating how Fas might play a role beyond T cells in controlling autoimmunity, not how TCR-induced death can be mediated via a non-Fas mechanism.

### Author Response

We agree with the reviewer's opinion, and have modified the sentence in question to emphasize that these data suggest Fas is less important for killing activated T cells in the context of repeated antigen restimulation than previously presumed. The next sentence provides further detail on this point.

4. You need to confirm that the SM1/23 antibody is saturating. Have you tried a dose-response where it flattens below 100%? Does this adequately address the issue of saturating antibody, as PBL were used in both cases.

### Author Response

We have confirmed that use of SM1/23 Fas blocking Ab is saturating at the 1 μg/ml dose used throughout the study, both in primary human T cells as well as Jurkat T cell lines. Adding more than 1 μg/ml did not provide additional protection.

5. In reference to sentence on pg 10 ("IL-2 signaling *normally *destabilizes BIM mRNA or promotes BIM protein degradation via Raf/ERK or phosphoinositide kinase 3 (PI-3K) signaling pathways.") Is this the right word or would "alone" be better?

### Author Response

Per the reviewer's suggestion, we have modified the sentence to read "IL-2 signaling alone can destabilize BIM mRNA..."

6. In reference to Bim knockdown in CD8^+ ^T cells (Figure 3B, Additional File 3): Could you titrate your siRNA so that you get a similar level of residual Bim in CD8s and see if you get the same compromise in apoptosis rescue?

### Author Response

We appreciate the reviewer's suggestion, and will try this approach in subsequent studies. On the other hand, our experience indicates that adding more siRNA does not improve BIM knockdown and may lead to off-target effects, which is why we did not pursue this further in the context of the current study.

7. Insert "T cell" on pg 13 to clarify the term "cross-tolerance".

### Author Response

The term is added as suggested, thank you.

## Supplementary Material

Additional File 1 **Blockade of Fas-induced apoptosis in SM1/23 treated PBL**. Activated PBL were pre-treated with 1 ug/ml SM1/23 Ab prior to addition of increasing amounts of APO1.3 Ab. Percent cell loss was calculated 24 h later by PI exclusion in triplicate. Click here for file

Additional File 2 **Bim siRNA effectively suppresses restimulation-induced BIM expression in activated T cells**. Activated human PBL, purified CD4+ and CD8+ T cells were transfected with nonspecific (NS) or Bim-specific siRNA and rested for 4 days. Lysates were made from cells left untreated (0 h) or restimulated for 8 h with 100 ng/ml OKT3. Knockdown of protein expression was confirmed by immunoblotting. Click here for file

Additional File 3 **Knockdown of FADD or Bim expression results in partial resistance to TCR-induced death**. Activated human PBL were transfected with nonspecific (NS), FADD-specific, or Bim-specific siRNA, rested for 4 days, and then restimulated for 24 h with increasing doses of OKT3 in the presence or absence of Fas blocking Ab (SM1/23). Percent cell loss was calculated in triplicate by PI exclusion (left panel). Knockdown of protein expression was confirmed by immunoblotting in whole lysates 4 days post-transfection (right panel). Click here for file

Additional File 4 **Knockdown of PUMA results in partial resistance to TCR-induced death**. Activated human PBL were transfected with nonspecific (NS), Puma-specific, or Bim-specific siRNA, rested for 4 days, and then restimulated for 24 h with increasing doses of OKT3 in the presence or absence of Fas blocking Ab (SM1/23). Percent cell loss was calculated in triplicate by PI exclusion (left panel). Knockdown of protein expression was confirmed by immunoblotting in whole lysates 4 days post-transfection (right panel). Click here for file

Additional File 5 **BIM induction in CD4^+ ^vs CD8^+ ^human T cells**. CD4^+ ^and CD8^+ ^T cells were purified from activated human PBL and restimulated with OKT3 for the indicated times. Whole cell lysates were prepared, separated by SDS-PAGE, and immunoblotted for BIM isoforms or BCL-2 expression. β-actin serves as a loading control. Click here for file

Additional File 6 **BIM siRNA impairs IL-2 withdrawal apoptosis in both CD4^+ ^and CD8^+ ^T cells**. Purified CD4^+ ^and CD8^+ ^T cells were transfected with nonspecific (NS) or Bim-specific siRNA and rested 24 hrs in IL-2. IL-2 was removed by thorough washing, and percent cell loss was calculated 72 and 96 hrs later by PI exclusion (left panel). The percent of apoptosis inhibition afforded by BIM siRNA (relative to NS) is graphed in the right panel. Click here for file

Additional File 7 **Impaired Fas-induced apoptosis in ALPS Ia patients**. Activated human PBL from normal control donors (NC1, NC2), or 6 ALPS type Ia patients were treated with increasing doses of APO1.3 mAb for 24 h. Percent cell loss was calculated in triplicate by PI exclusion. Click here for file

Additional File 8 **ALPS Type IV patient T cells express functional FAS**. (A) Activated PBL from a normal control donor (NC, open histogram), an ALPS type Ia patient (gray), or ALPS type IV (P58, black) were stained with FITC-conjugated anti-CD95 or isotype control Ab (dashed line) and analyzed by flow cytometry. (B) Activated PBL from a normal donor (NC), an ALPS type IV patient (P58) and an ALPS type Ia patient were treated with 20 or 200 ng/ml APO1.3 mAb plus 200 ng/ml Protein A. Percent cell loss was calculated in triplicate by PI exclusion. Click here for file
