# Supplementary figures and images for: Critical role for BIM in T cell receptor restimulation-induced death
Source: Biol Direct. 2008 Aug 20;3:34. doi: 10.1186/1745-6150-3-34 (PMC2529272; doi:10.1186/1745-6150-3-34)

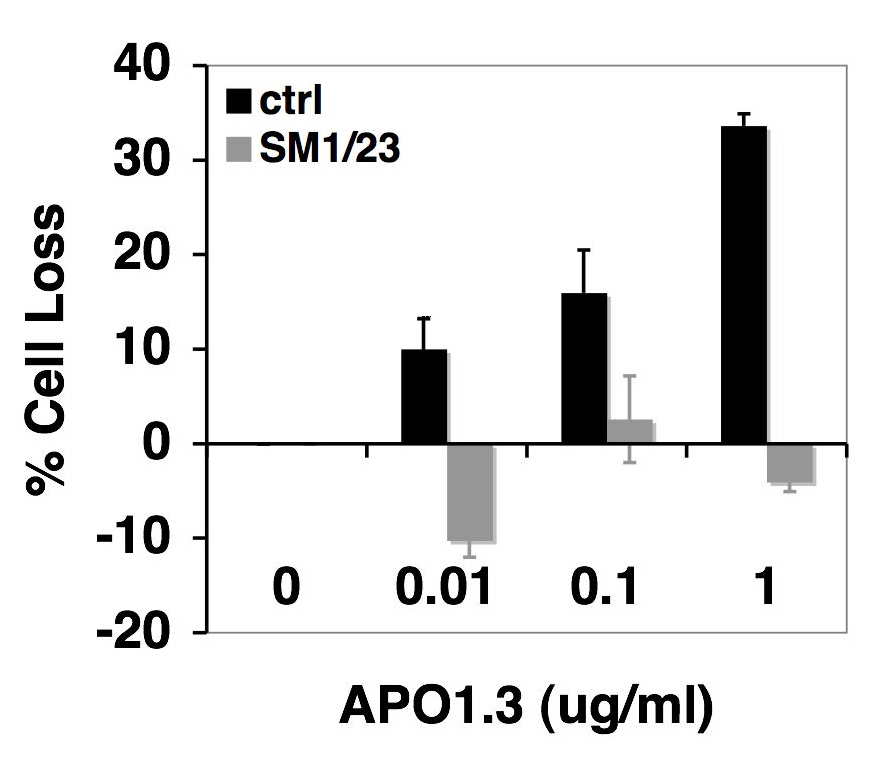

Supplement: Additional File 1 — Blockade of Fas-induced apoptosis in SM1/23 treated PBL. Activated PBL were pre-treated with 1 ug/ml SM1/23 Ab prior to addition of increasing amounts of APO1.3 Ab. Percent cell loss was calculated 24 h later by PI exclusion in triplicate. [file 1745-6150-3-34-S1.jpeg]

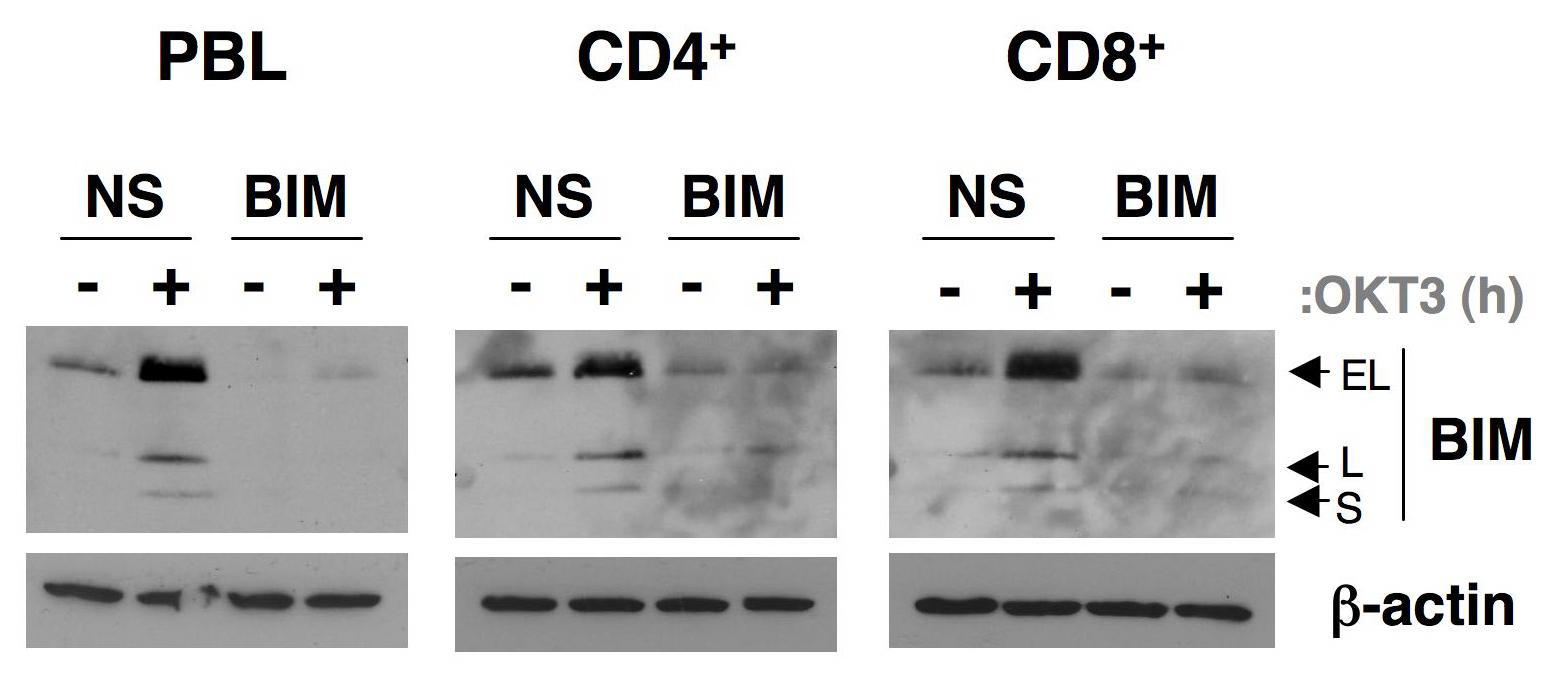

Supplement: Additional File 2 — Bim siRNA effectively suppresses restimulation-induced BIM expression in activated T cells. Activated human PBL, purified CD4+ and CD8+ T cells were transfected with nonspecific (NS) or Bim-specific siRNA and rested for 4 days. Lysates were made from cells left untreated (0 h) or restimulated for 8 h with 100 ng/ml OKT3. Knockdown of protein expression was confirmed by immunoblotting. [file 1745-6150-3-34-S2.jpeg]

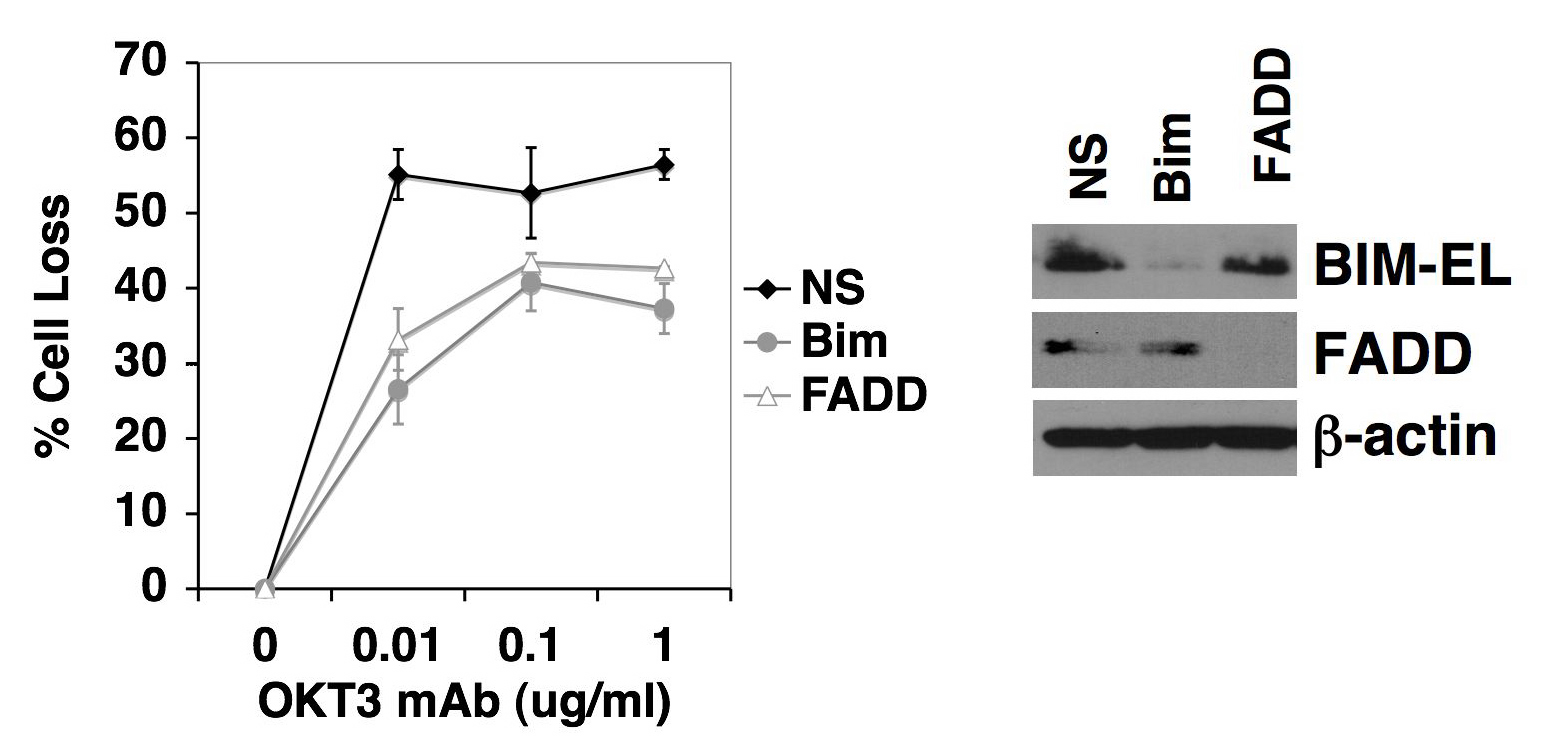

Supplement: Additional File 3 — Knockdown of FADD or Bim expression results in partial resistance to TCR-induced death. Activated human PBL were transfected with nonspecific (NS), FADD-specific, or Bim-specific siRNA, rested for 4 days, and then restimulated for 24 h with increasing doses of OKT3 in the presence or absence of Fas blocking Ab (SM1/23). Percent cell loss was calculated in triplicate by PI exclusion (left panel). Knockdown of protein expression was confirmed by immunoblotting in whole lysates 4 days post-transfection (right panel). [file 1745-6150-3-34-S3.jpeg]

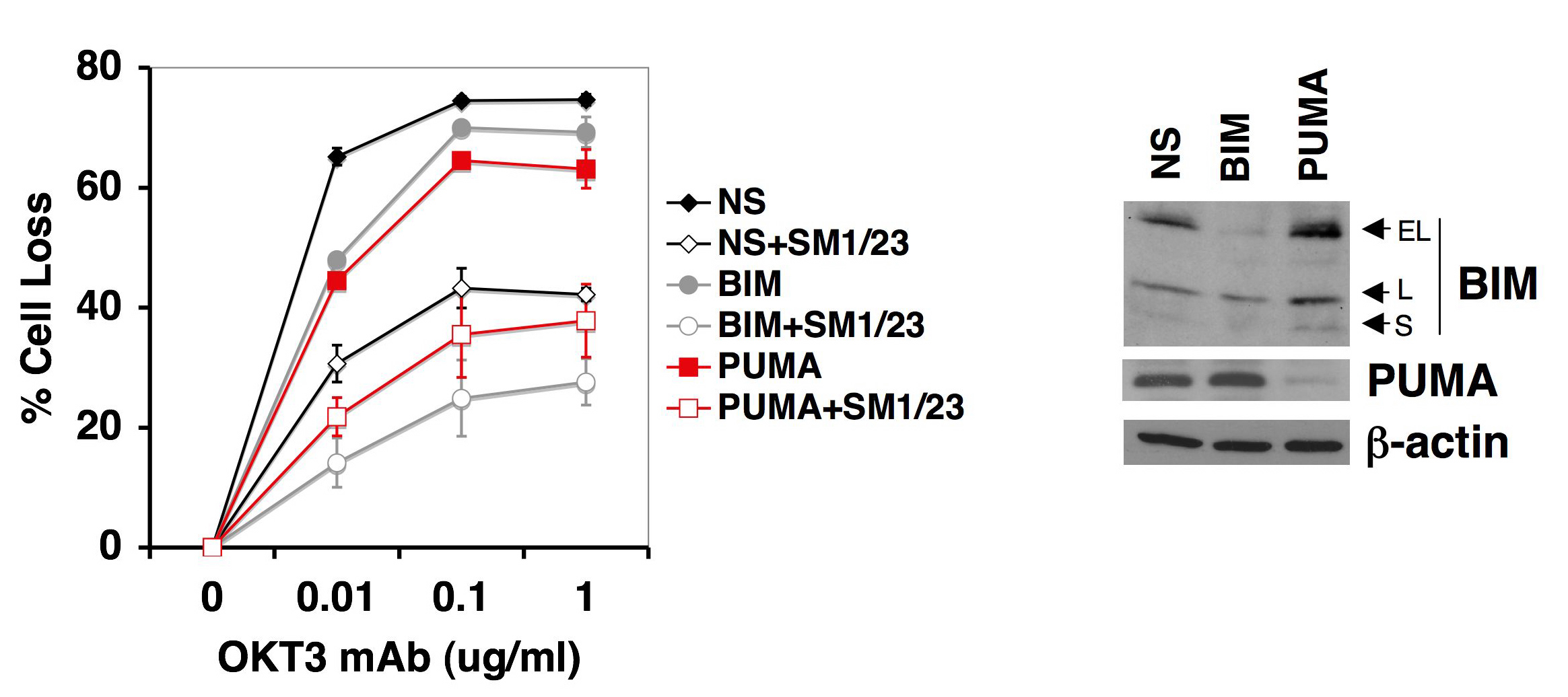

Supplement: Additional File 4 — Knockdown of PUMA results in partial resistance to TCR-induced death. Activated human PBL were transfected with nonspecific (NS), Puma-specific, or Bim-specific siRNA, rested for 4 days, and then restimulated for 24 h with increasing doses of OKT3 in the presence or absence of Fas blocking Ab (SM1/23). Percent cell loss was calculated in triplicate by PI exclusion (left panel). Knockdown of protein expression was confirmed by immunoblotting in whole lysates 4 days post-transfection (right panel). [file 1745-6150-3-34-S4.jpeg]

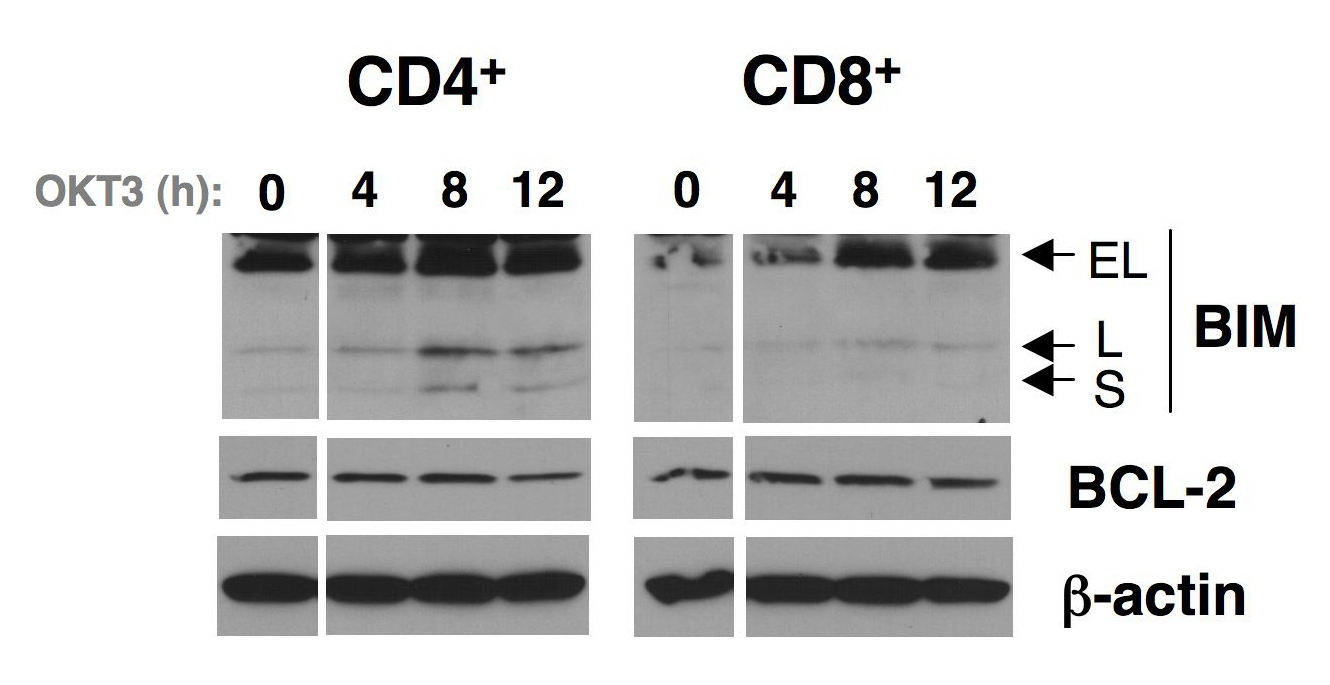

Supplement: Additional File 5 — BIM induction in CD4+ vs CD8+ human T cells. CD4+ and CD8+ T cells were purified from activated human PBL and restimulated with OKT3 for the indicated times. Whole cell lysates were prepared, separated by SDS-PAGE, and immunoblotted for BIM isoforms or BCL-2 expression. β-actin serves as a loading control. [file 1745-6150-3-34-S5.jpeg]

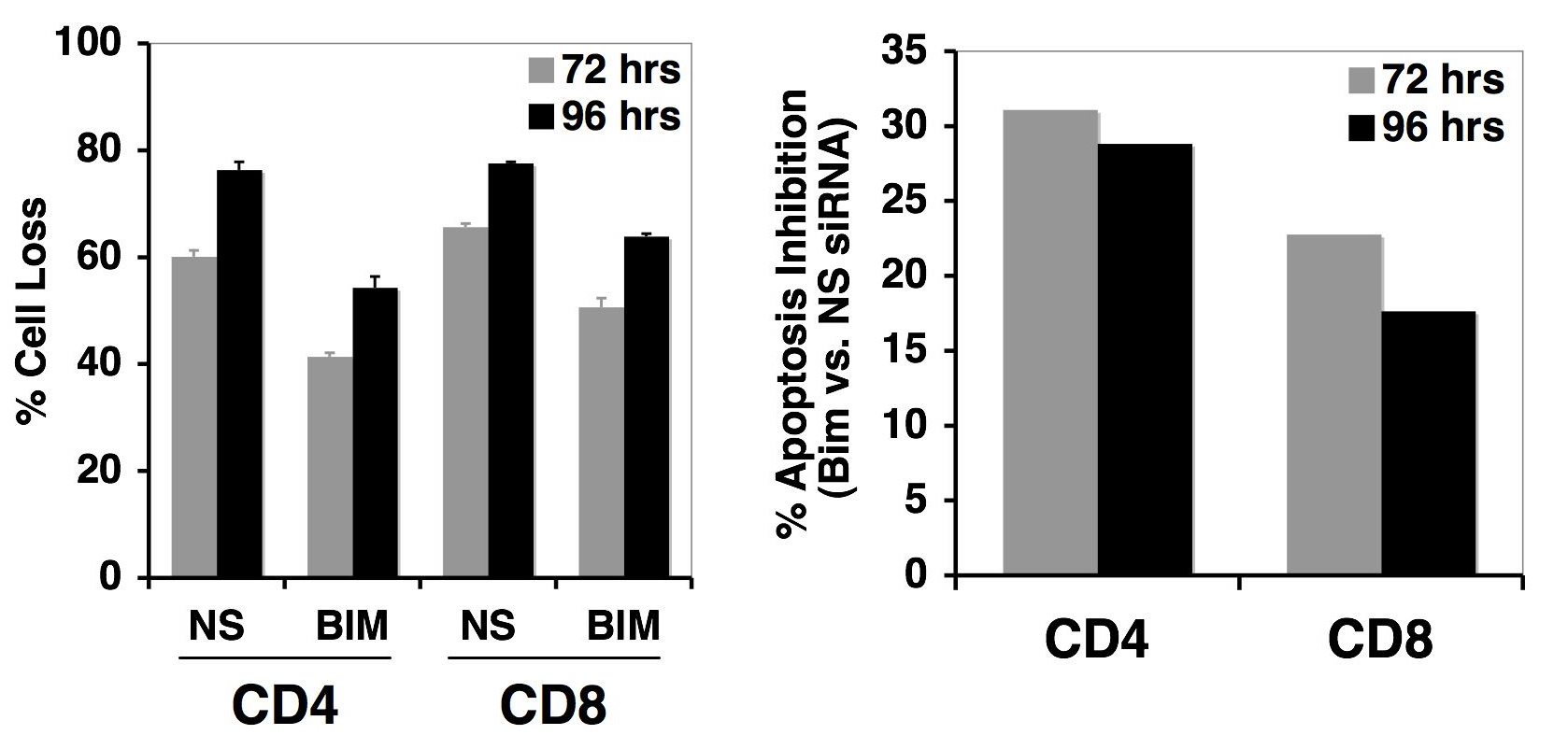

Supplement: Additional File 6 — BIM siRNA impairs IL-2 withdrawal apoptosis in both CD4+ and CD8+ T cells. Purified CD4+ and CD8+ T cells were transfected with nonspecific (NS) or Bim-specific siRNA and rested 24 hrs in IL-2. IL-2 was removed by thorough washing, and percent cell loss was calculated 72 and 96 hrs later by PI exclusion (left panel). The percent of apoptosis inhibition afforded by BIM siRNA (relative to NS) is graphed in the right panel. [file 1745-6150-3-34-S6.jpeg]

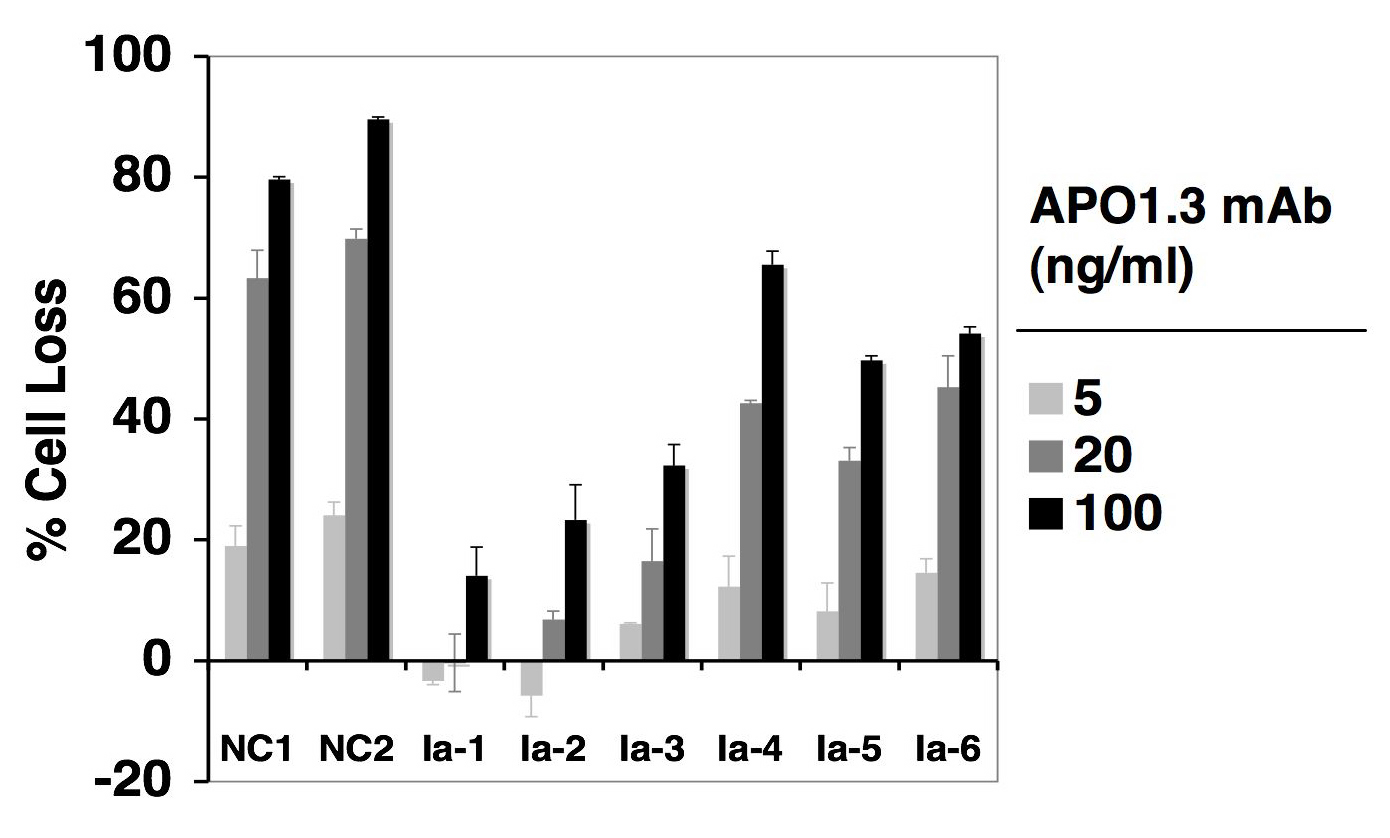

Supplement: Additional File 7 — Impaired Fas-induced apoptosis in ALPS Ia patients. Activated human PBL from normal control donors (NC1, NC2), or 6 ALPS type Ia patients were treated with increasing doses of APO1.3 mAb for 24 h. Percent cell loss was calculated in triplicate by PI exclusion. [file 1745-6150-3-34-S7.jpeg]

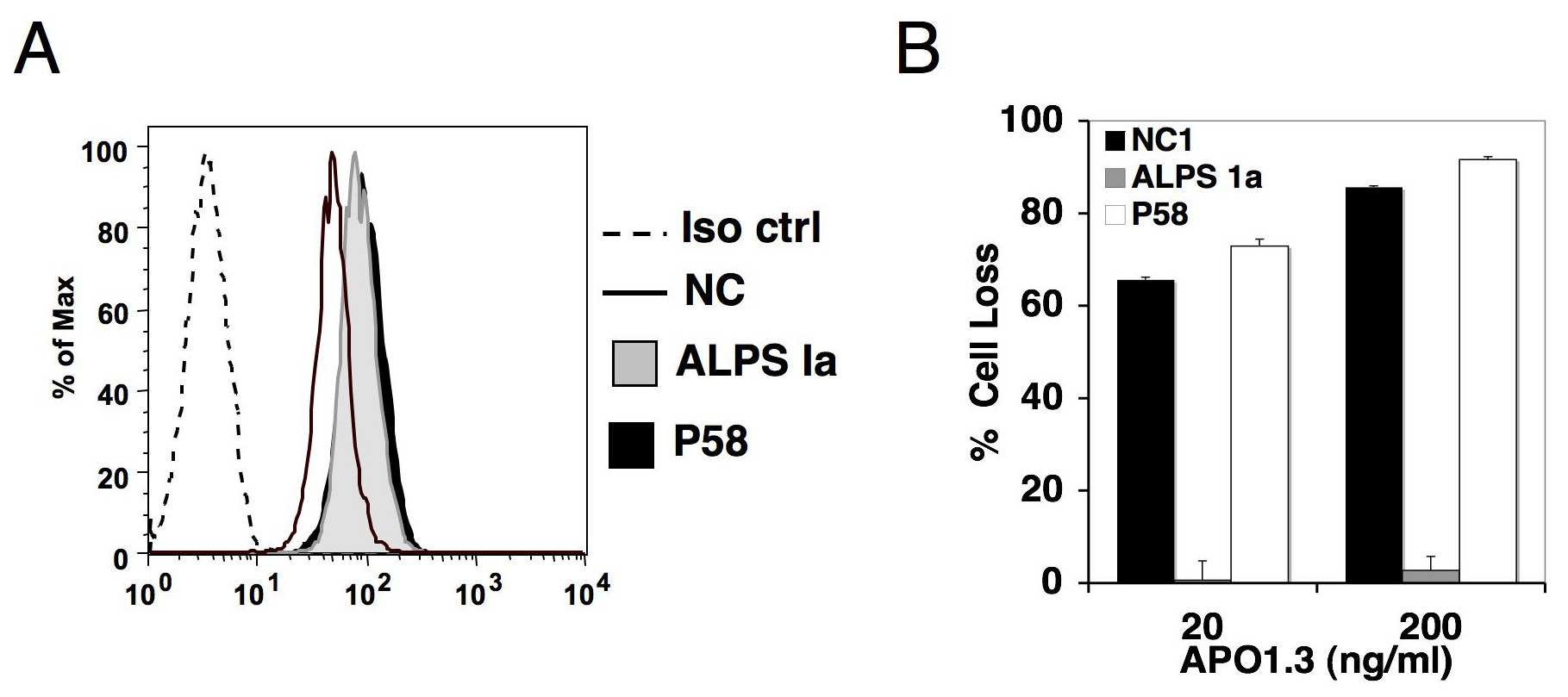

Supplement: Additional File 8 — ALPS Type IV patient T cells express functional FAS. (A) Activated PBL from a normal control donor (NC, open histogram), an ALPS type Ia patient (gray), or ALPS type IV (P58, black) were stained with FITC-conjugated anti-CD95 or isotype control Ab (dashed line) and analyzed by flow cytometry. (B) Activated PBL from a normal donor (NC), an ALPS type IV patient (P58) and an ALPS type Ia patient were treated with 20 or 200 ng/ml APO1.3 mAb plus 200 ng/ml Protein A. Percent cell loss was calculated in triplicate by PI exclusion. [file 1745-6150-3-34-S8.jpeg]
